# The "cut-in patch-out" technique for Pancoast tumor resections results in postoperative pain reduction: a case control study

**DOI:** 10.1186/s13019-014-0163-z

**Published:** 2014-09-30

**Authors:** Daniel J Weber, Ikenna C Okereke, Thomas J Birdas, DuyKhanh P Ceppa, Karen M Rieger, Kenneth A Kesler

**Affiliations:** Division of Cardiothoracic Surgery, Department of Surgery, Indiana University Melvin and Bren Simon Cancer Center, Indianapolis, IN USA; Division of Thoracic Surgery, The Rhode Island and Miriam Hospitals of Warren Alper Medical School of Brown University, Providence, RI USA; Department of Surgery, Cardiothoracic Division, Indiana University School of Medicine, 545 Barnhill Dr, Emerson Hall 215, Indianapolis, 46202 IN USA

**Keywords:** Pancoast tumor, Lung cancer, Chest wall, Pain

## Abstract

**Background:**

Since 2001 we have utilized a novel surgical approach for Pancoast tumors in which lobectomy and mediastinal lymph node dissection are performed directly though the chest wall defect. The defect is then patched at the completion of the procedure ("cut-in patch-out") thereby avoiding a separate thoracotomy with rib spreading. We undertook a study to compare outcomes of this novel "cut-in patch-out" technique with traditional thoracotomy for patients with Pancoast tumors.

**Methods:**

We retrospectively identified 41 patients undergoing surgical resection of Pancoast tumors requiring en-bloc removal of at least 3 ribs at our institution from 1999 to 2012. Surgery was accomplished by either a "cut-in patch-out" technique (n = 25) or traditional posterolateral thoracotomy and separate chest wall resection (n = 16). Multiple variables including patient demographics, neoadjuvant therapy, extent of resection, and pathology were analyzed with respect to outcomes from morbidity, narcotic use, and oncologic perspectives.

**Results:**

Baseline demographics, neoadjuvant therapy, and perioperative factors including extent of surgery, complete resections (R0), nodal status and lymph node number, morbidity, and mortality were similar between the two groups. The mean duration of out-patient narcotic use was significantly lower in the "cut-in patch-out" group compared to the thoracotomy group (80.6 days ± 62.4 vs. 158.2 days ± 119.2, p < 0.01). Using multivariate regression analysis, the traditional thoracotomy technique (OR 7.72; p = 0.01) was independently associated with prolonged oral narcotic requirements (>100 days). Additionally, five year survival for the "cut-in patch-out" group was 48% versus the traditional group at 12.5% (p = 0.04).

**Conclusions:**

Compared with a traditional thoracotomy and separate chest wall resection approach for P-NSCLC, a "cut-in patch-out" technique offers an alternative approach that appears to have at least oncologic equivalence while decreasing pain. We have more recently adapted this technique to select patients with pulmonary neoplasms involving chest wall invasion and believe further investigation is warranted.

**Electronic supplementary material:**

The online version of this article (doi:10.1186/s13019-014-0163-z) contains supplementary material, which is available to authorized users.

## Background

Although initially characterized by a radiologist, Henry Pancoast, in 1924, surgical resection of a superior sulcus or Pancoast tumor was not attempted until the 1950s [1],[2]. It is estimated that 5% of all non-small cell lung cancers (NSCLCs) require chest wall resection with approximately 20% of these cases considered to be Pancoast tumors (P-NSCLC) involving the apical ribs [3]. Current recommendations for P-NSCLC patients with localized disease include neoadjuvant chemoradiation therapy followed by surgical resection [4]. Surgery for P-NSCLC is particularly challenging. Traditionally, a posterolateral thoracotomy has been initially performed to both divide pulmonary hilar structures and perform mediastinal lymph node dissection followed by resection of the involved chest wall [5]. It has been well established however that significant morbidity can be associated with rib spreading during thoracotomy including impairment of pulmonary mechanics as well as prolonged return of functionality [6]-[8]. Such concerns are further compounded for those patients undergoing surgery for P-NSCLC tumors who require a chest wall resection in addition to a thoracotomy. It is therefore not surprising that P-NSCLC patients often experience postoperative pulmonary complications as well as require prolonged narcotic requirement for pain management.

Since 2001 we have utilized a novel surgical approach for P-NSCLC requiring en-bloc removal of at least 3 ribs where lobectomy and mediastinal lymph node dissection are performed though the defect after chest wall resection. The defect is patched at the completion of the procedure ("cut-in patch-out") thereby avoiding a separate thoracotomy with rib spreading. The aim of this study was to compare short and long term outcomes between P-NSCLC patients undergoing this novel technique and a traditional posterolateral thoracotomy approach.

## Methods

### Study design

Under the Indiana University School of Medicine Institutional Review Board approval, a query of an institutional database was undertaken. Forty-one patients undergoing surgical resection of a P-NSCLC requiring en-bloc removal of at least 3 ribs from 1999 to 2012 were identified. Since 2001, 25 patients were identified who underwent a novel "cut-in patch-out" approach. Sixteen patients underwent resection of a P-NSCLC by a traditional posterolateral thoracotomy and separate chest wall resection who served as controls. Patients with anterior based apical tumors requiring a partial sternotomy approach (n = 6) and P-NSCLC who required en bloc removal of only 2 or fewer ribs (n = 13) were excluded from this study.

Patient demographic data including age, gender, neoadjuvant chemotherapy/radiation therapy, and co-morbidities were collected. Operative and pathologic variables including tumor size, histology, stage, number of ribs resected, estimated blood loss, type of surgical resection (R0, R1, and R2), and number of mediastinal lymph nodes harvested were collected. Short term post-operative data including duration of intravenous narcotics, length of hospital stay, morbidity (pulmonary and non-pulmonary morbidity) and mortality were recorded. Survival data was verified with the Social Security Death Index. Long-term postoperative data including duration of daily narcotic use following discharge were collected and verified by a statewide narcotic tracking system. This system, under the auspices of the state government, provides an online database of all prescribed controlled substances, including narcotics. Finally, status at time of last follow up was obtained.

### Operative procedure

**Figure 1:**
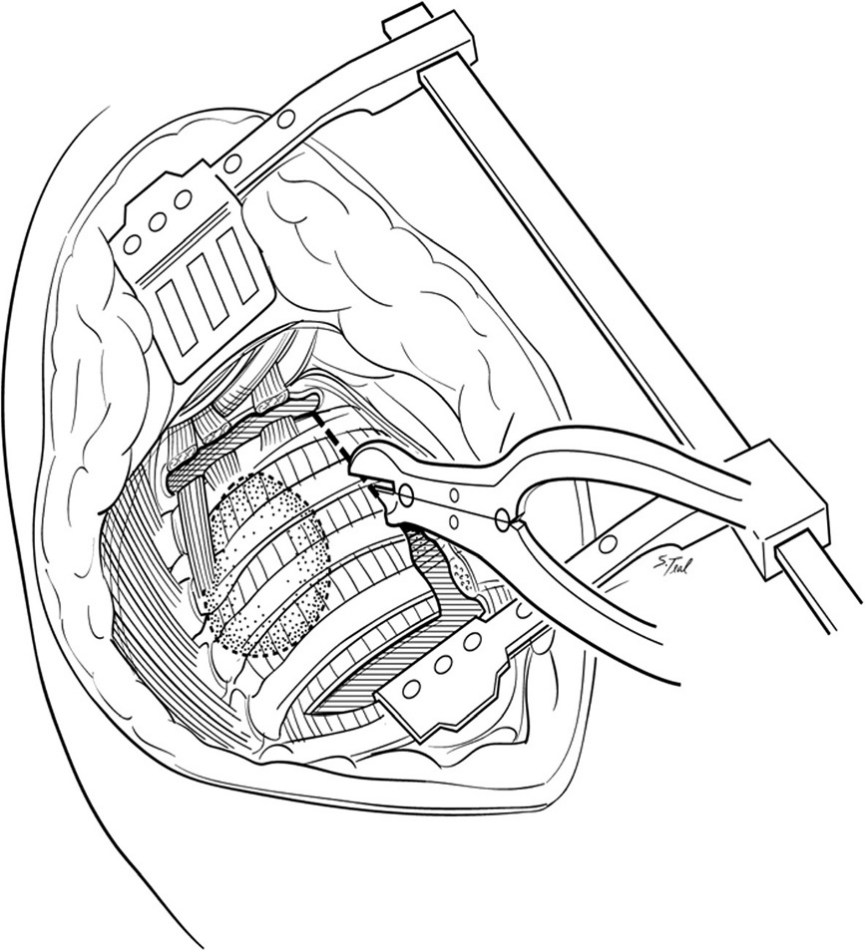
**The "cut-in patch-out" technique.** The pleural space is initially entered in the lowest tumor free interspace typically 3 to 5 cm anterior and inferior to the tumor location, as determined by preoperative CT scan, then extended posteriorly. The anterior aspect of the chest resection was then performed 3 to 5 cm anterior to the tumor location facilitated by upward scapular retraction. A one cm segment of rib is excised anteriorly to improve chest wall mobility.

**Figure 2:**
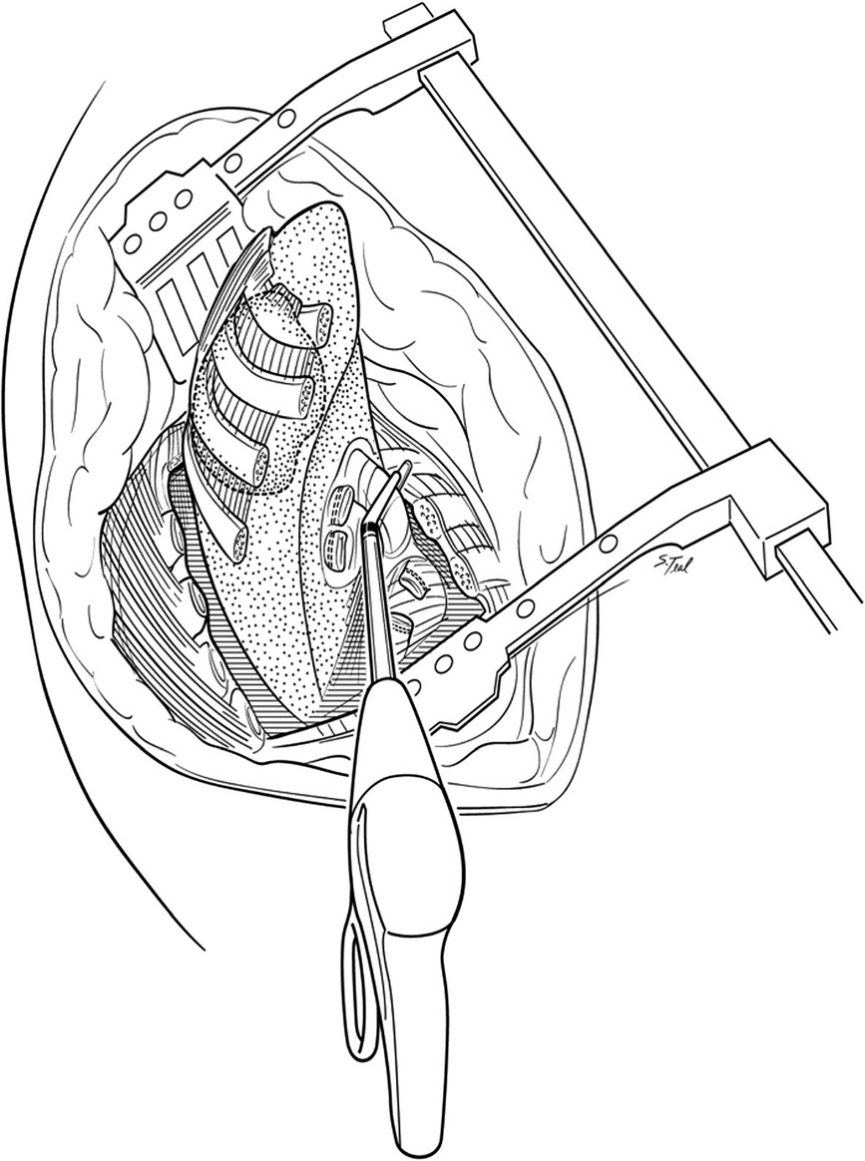
**Rib disarticulation and hilar dissection.** The ribs are disarticulated posteriorly from their respective transverse processes and vertebral bodies. After chest wall resection is complete, hilar dissection including division of lobar vessels and airways for lobectomy along with complete peribronchial and mediastinal lymph node dissection is performed through the chest wall defect itself facilitated with the use of endoscopic stapling devices.

**Figure 3:**
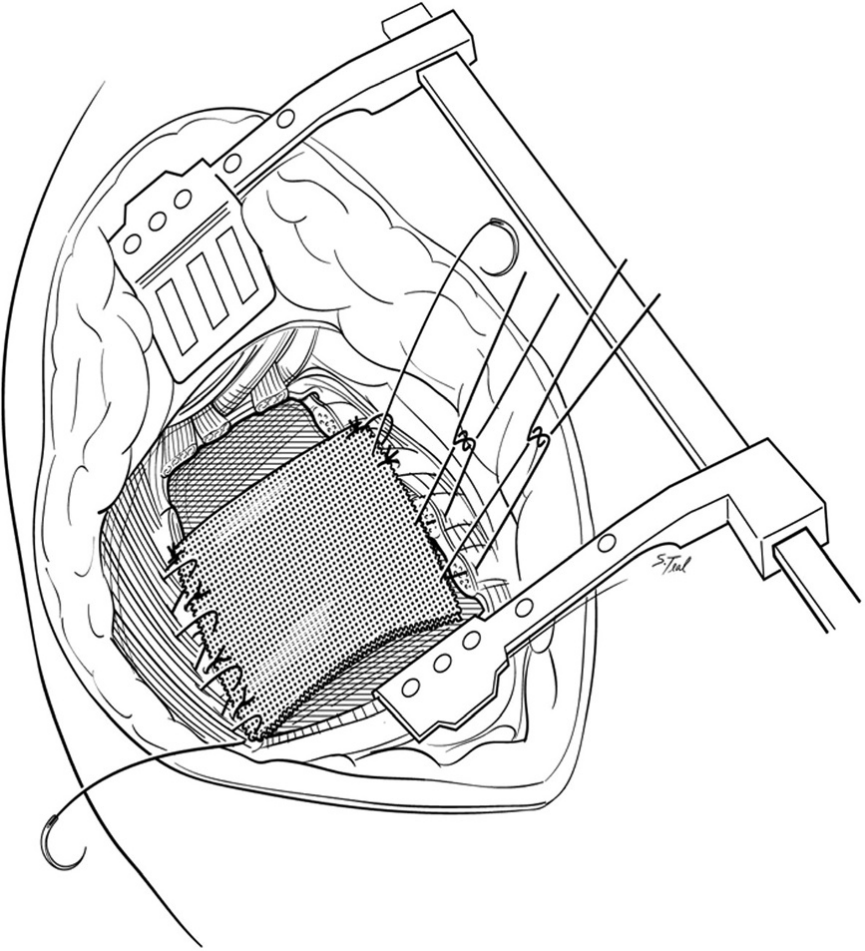
**Chest wall reconstruction.** After the specimens are removed, the chest wall defect is closed with a double layer of a Vicryl mesh. The mesh is initially secured to the transverse processes then to rib edges with interrupted 0-polyproplene sutures reinforced with a running looped 0-polydioxanone suture. Typically the first rib and first transverse process are not included in the patch to avoid contact with the brachial plexus or subclavian vessels.

All surgery was performed at Indiana University Melvin and Bren Simon Cancer Center. Traditional resections were performed through an extended poster lateral thoracotomy incision with a separate chest wall resection as described by Paulson and Shaw [9]. These procedures typically involved a 5^th^ interspace thoracotomy with rib spreading for hilar dissection and lobectomy along with complete peribronchial and mediastinal lymph node dissection. Apical en bloc chest wall resection was typically accomplished after hilar dissection in these cases. After 2001, a majority of patients with P-NSCLC underwent resection using a "cut-in patch-out" technique which did not involve a separate thoracotomy. With the "cut-in patch-out" technique, an extended posterolateral incision was also utilized. In these cases however, the pleural space was initially entered in the interspace estimated to be 3 to 5 cm anterior and inferior to the tumor location, as determined by preoperative CT scan. The tumor was palpated and, if clear of the interspace entry site, the interspace was opened posteriorly to the costovertebral angle. The anterior aspect of the chest resection was then performed 3 to 5 cm anterior to the tumor location, again confirmed visually or by palpation. A one cm segment of rib was excised anteriorly to improve chest wall mobility which was facilitated by upward scapular retraction (Figure 1). The superior border of the resection was established at the level of the first interspace if the first rib could be spared or more commonly, the soft tissues superior up to the first rib including scalene muscles with careful attention to spare the subclavian vessels and brachial plexus when oncologically feasible. Also when possible, the T1 nerve root to the lower trunk of the brachial plexus was also spared to maintain ulnar nerve function. The ribs were then disarticulated posteriorly from their respective transverse processes and vertebral bodies carefully occluding intercostal vessels and nerves when encountered. After chest wall resection was complete, lobectomy and peribronchial and mediastinal lymph node dissections were performed through the chest wall defect itself (Figure 2). Ligation and division of hilar structures was facilitated with the use of endoscopic stapling devices designed for thoracoscopic lobectomy. Commonly the stapling devices were placed through the chest wall defect itself although occasionally if the chest wall defect was limited, a separate small incision in the inferior chest was necessary for stapler application. After the specimens are removed, the chest wall defect was closed with a double layer of a Vicryl mesh (Ethicon Inc, Somerville, NJ) (Figure 3). Mesh was secured to the transverse processes and then to the rib edges and with interrupted 0-polyproplene sutures and reinforced with a running looped 0-polydioxanone suture. In both approaches, two standard chest tube drains were placed in the anterior and posterior pleural space. Additionally a soft tissue drain was typically placed between the Vicryl mesh and scapula.

### Statistical analysis

All statistical analysis was performed with Statistical Package for the Social Science (SPSS, Chicago, IL) version 20.0 for Windows. Values are presented as means with standard deviations unless otherwise specified. Continuous variables were compared using student t-test while categorical variables were compared with the chi squared. Survival and recurrence analysis was performed using the Kaplan-Meier method. A multivariate regression analysis for prolonged duration of oral narcotics (>100 days) was adjusted for narcotic use preoperatively, the number of ribs resected, estimated blood loss, length of hospital stay, and surgical technique. All tests were two-sided, with 0.05 serving as the level of significance.

## Results

In total, 25 patients underwent the "cut-in patch-out" approach while 16 patients underwent a traditional posterolateral thoracotomy and separate chest wall resection. Patient characteristics are presented in Table 1. The two groups were similar with regards to age, gender, race, preoperative narcotic and tobacco use. The majority of P-NSCLC in both groups involved the left lung and chest wall. Adenocarcinoma was the most prevalent pathology in both groups. Due to the increasing use of induction therapy over time, more patients received neoadjuvant therapy who underwent the "cut-in patch-out" technique (96.0%) as compared to the traditional group (87.5%) although this did not reach statistical significance.

**Table 1:** **Patient and operative characteristics**

Characteristic	Cut-in patch out (n = 25)	Thoracotomy (n = 16)	***p***value
Mean age (SD)	56.9 (±9.4)	57.3 (±9.6)	0.90
Female (%)	15 (60.0%)	8 (50.0%)	0.76
Race: White (%)	20 (80.0%)	11 (68.8%)	0.66
Mean Pack Years (SD)	48.6 (±26.4)	44.2 (±29.4)	0.62
Taking Oral Narcotics Preoperatively	3 (12.0%)	4 (25.0%)	0.40
Neoadjuvant Treatment			
Radiation Only (%)	2 (8.0%)	2 (12.5%)	0.64
Chemoradiation (%)	22 (88.0%)	12 (75.0%)	0.40
Location: Left (%)	19 (73.1%)	11 (68.8%)	0.88
Pathology			
Adenocarcinoma (%)	13 (52.0%)	8 (50.0%)	0.98
Squamous (%)	9 (36.0%)	7 (43.8%)	0.75
Other (%)	3 (12.0%)	1 (6.3%)	0.98
Epidural pain catheter (%)	14 (56.0%)	9 (56.3%)	0.98
Estimated Blood Loss (SD)	442 (±223)	423 (±256)	0.83
Mean ribs resected (SD)	3.65 (±0.83)	3.38 (±0.51)	0.25
Mean tumor Size in cm (SD)	4.20 (±3.03)	4.71 (±2.14)	0.56
Mean number of nodes sampled (SD)	14.2 (±5.1)	14.8 (±6.6)	0.75
Positive lymph nodes (%)	2 (8.0%)	4 (25.0%)	0.19
R0 resection	23 (92.0%)	14 (87.5%)	0.64
R1 resection	2 (8.0%)	2 (12.5%)	
Final Pathology Staging			
≤ T2	3 (12.0%)	2 (12.5%)	0.96
T3	10 (40.0%)	6 (33.3%)	0.87
T4	12 (48.0%)	8 (50.0%)	0.90
Vertebral Body	9 (40.0%)	6 (37.5%)	0.93
Subclavian Vessels	3 (12.0%)	2 (12.5%)	0.96
N0	23 (92.0%)	11 (68.8%)	0.13
N1	1 (4.0%)	1 (6.3%)	0.74
N2	1 (4.0%)	3 (18.8%)	0.31

Categorical data presented as a number (%) while continuous data presented as mean ± standard deviation unless otherwise stated. p-values represent with either independent sample t-test or chi-squared as dictated by data type.

Perioperative factors were also similar between both groups (Table 1). All patients underwent anatomic upper lobectomies with complete peribronchial and mediastinal lymph node dissections. No differences were appreciated in the utilization of thoracic epidural catheters, estimated blood loss, or the number of ribs resected. Other factors such as tumor size, number of lymph nodes sampled, nodal status, and R0 resection rates were similar. While there was a trend for higher N2 disease in the traditional group, final pathologic staging was statistically similar in both groups. In terms of T4 tumor invasion, the most common site was the vertebral body in both groups.

Postoperative outcomes are presented in Table 2. The duration of intravenous narcotics was similar for both groups, with a mean of 6.3 days for the "cut-in patch-out" group and 6.0 days for the traditional thoracotomy group. Additionally, average lengths of hospital stay were also statistically similar at 13.1 days and 12.1, days respectively. While complication rates were slightly higher in the traditional group, none of these reached statistically significance. In particular, one patient in each group developed a wound infection, neither or which required surgical intervention. Pulmonary complications including pneumonia, reintubation, tracheostomy, pulmonary embolism, and bronchopleural fistulae were similar across both groups. There was no operative mortality with one death in both groups within 90 days of surgery.

**Table 2:** **Post-operative outcomes**

Outcome	Cut-in patch out (n = 25)	Thoracotomy (n = 16)	***p***value
Length of stay in days (SD)	13.1 (±7.42)	12.6 (±6.11)	0.82
Mean days on IV narcotics (SD)	6.2 (±3.75)	6.0 (±3.39)	0.86
Morbidity	7 (28.0%)	4 (25.0%)	0.83
Wound Infection	1 (4.0%)	1 (6.3%)	0.74
Pneumonia	4 (16.0%)	3 (18.8%)	0.82
Reintubation	5 (24.0%)	5 (31.3%)	0.65
Tracheostomy	3 (12.0%)	4 (25.0%)	0.51
Pulmonary Embolism	2 (8.0%)	1 (6.3%)	0.83
Bronchopleural Fistula	1 (4.0%)	1 (6.3%)	0.74
Mortality			
30-day mortality	0 (0%)	0 (0%)	1.00
90-day mortality	1 (4.0%)	1 (6.3%)	0.97
Mean days on oral narcotics (SD)	80.6 (±62.4)	158.2 (±119.2)	<0.01
Alive at 5 years (%)	12 (48.0%)	2 (12.5%)	0.04
Recurrence at 5 years (%)	6 (24.0%)	10 (62.5%)	0.02
Site of Recurrence			
Local (%)	2 (8.0%)	3 (18.8%)	0.36
Distant (%)	4 (16.0%)	7 (43.8%)	0.07

Categorical data presented as a number (%) while continuous data presented as mean ± standard deviation unless otherwise stated. p-values represent with either independent sample t-test or chi-squared as dictated by data type.

**Figure 4:**
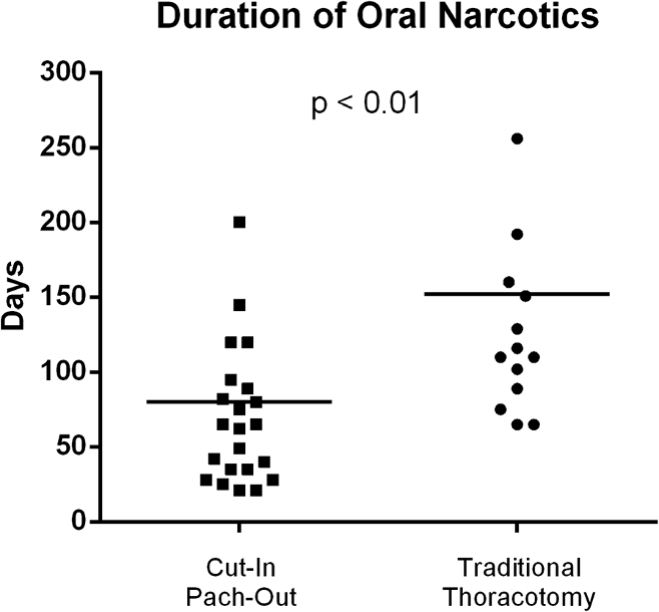
**Scatter-plot distribution demonstrating length of outpatient oral narcotic requirements of the "cut-in patch-out" versus traditional techniques.** Bar represents mean days of oral narcotic use.

Daily outpatient narcotic use was significantly lower in the "cut-in patch-out" group (80.6 days versus 158.2 days, p < 0.01) (Figure 4). With respect to long-term oncologic outcomes the "cut-in patch-out" group had lower five-year recurrence rates (24.0% versus 62.5%, p = 0.02) and improved five-year survival rates (48.0% versus 12.5%, p = 0.04). Finally, systemic recurrence was more common than local recurrence in both groups.

Results of multivariate regression analysis to identify risk factors for prolonged oral narcotic use (>100 days) is given in Table 3. In summary, although there was a trend towards preoperative narcotic use predicting prolonged oral narcotic use, this did not reach statistical significance. Additionally, prolonged oral narcotic use was not associated with having more than 3 ribs resected, estimated blood loss, or length of stay. However, the traditional technique was strongly associated with prolonged oral narcotic use (OR 8.28, p = 0.01).

**Table 3:** **Risk factors for duration of oral narcotics >100 days**

	Odds ratio (95% C.I.)	p-value
Taking Narcotics Preoperatively	1.19 (0.02-8.67)	0.38
More than 3 Ribs Resected	1.14 (0.15-2.36)	0.18
Estimated Blood Loss	1.01 (0.98-1.03)	0.13
Length of Stay	1.08 (0.98-1.21)	0.42
Traditional Technique	8.28 (1.54-44.41)	0.01

Multiple regression analysis for prolonged oral narcotic use (>100 days) following P-NSCLC resection. CI = Confidence Interval.

## Discussion

The results of this study demonstrate that a "cut-in patch-out" approach to P-NSCLC can offer a reduction in postoperative pain when compared with a traditional approach. Although not seen in the immediate post-operative course with intravenous narcotic use, the benefit became apparent after discharge analyzing the duration of oral narcotic requirement by both univariate and in a multivariate model. From an oncologic standpoint, the number of mediastinal lymph nodes retrieved and surgical margin status were similar between the two groups. More importantly, five-year recurrence rates and survival were both improved in patients undergoing the "cut-in patch-out" technique suggesting this approach offers at least an equivalent oncologic outcome.

A "cut-in patch-out" type of surgical approach to P-NSCLC, was initially reported by Kent et al. from Memorial Sloan-Kettering Cancer Center using a polytetraflouroethylene patch for chest wall reconstruction in 2004 [10]. We have been using this technique at our institution dating back to 2001. One limitation to this approach is that it is only feasible if at least 3 apical ribs are removed. Optimal exposure to the pulmonary hilum and mediastinal lymph nodes is however provided if 4 or more ribs are removed. We believe there is little if any downside with respect to additional pain or reduction of chest wall mechanics following upper lobectomy and removal of the upper 4 ribs to optimize exposure. In the less common scenario where only one or two ribs require removal, division of the hilar vessels and airway will usually require a separate thoracotomy or a VATS-assisted approach. The use of VATS for P-NSCLC tumors has been reported to have good preliminary results with cited advantages including the ability to assess the subclavian vessels and hilum minimally invasively [11]. Additionally, a recent report has demonstrated the feasibility of a VATS approach to perform a limited en bloc resection for a superior sulcus resection [12]. Specific advantages to a VATS approach for P-NSCLC need further study however.

There have been many options described for chest wall reconstruction after P-NSCLC resection including polypropylene and polytetrafluoroethylene prosthetic mesh [3],[4],[13]. Some authors have described using no patch reconstruction as the scapula typically covers the bony chest wall defect [10]. We have used a double layer Vicryl mesh, which does not require removal in case of a low-grade infection with the added advantage of stabilizing the cut ribs pending scar tissue replacement. No patients in our series required Vicryl mesh removal despite two patients developing overlying soft tissue infections which are prone to occur after radiation therapy and relative soft tissue devascularization after rib removal.

Although previous work has investigated the prevalence of post-thoracotomy pain, there is little information on pain after a chest wall resection. One retrospective analysis of thoracotomy patients by Keller et al. determined that 50% of those undergoing chest wall resections with thoracotomy developed significant post-thoracotomy pain compared with 11% of those undergoing thoracotomy without chest wall resection [14]. A more recent study used a post-operative questionnaire to assess quality of life after thoracotomy and found that only 16% of patients were using narcotics for pain control 3 months after surgery [6]. This appears to be somewhat lower that our study where 26.1% of patients in the "cut-in patch-out" group and significantly lower than 53.8% in the traditional thoracotomy group still requiring narcotics at 3 months. These discrepancies would not be unexpected as the pain associated with P-NSCLC surgery is undoubtedly worsened by chest wall resection as well as a separate rib spreading thoracotomy in the subset of patients undergoing traditional surgery. Of note, an advantage to this current study is that outpatient narcotic use was measured using a statewide electronic narcotic tracking program thereby reducing measurement bias.

The factors that have been shown to be associated with the best outcomes for P-NSCLC resections include R0 resections, absence of positive nodes, and anatomic lobar versus wedge pulmonary resections [15]. Although these variables were all similar between the groups, there was a trend towards higher number of positive N2 disease in the traditional thoracotomy group. This factor, along with a lower R0 resection rate and lower use of neoadjuvant therapy early in our series, may have contributed to reduced long-term survival in the traditional surgery group. Subsequent treatment guidelines set forth by the NCCN and ACCP have recommend neoadjuvant chemoradiation therapy followed by a consideration of surgery so it is not surprising that more patients undergoing the "cut in patch out" technique did receive neoadjuvant therapy. After trimodality therapy, five-year survival rates for patients with P-NSCLC have been reported to range between 41% and 59% [16]-[18].

There are several limitations to this study. First, this is a small retrospective series at a single institution. Although reduction in oral narcotic requirements were apparent in the "cut-in patch-out" group, larger and more uniform patient subsets are needed to determine differences from long-term oncologic perspectives including the ability to achieve tumor free margins. Another limitation is that although the majority of patients appeared to be taking similar narcotic doses, we were only able to determine the use of narcotics per se and could not obtain accurate dosage information. Additionally, although it appeared that non-narcotic pain medications such as non-steroidal anti-inflammatory drugs or gamma-aminobutyric acid analogs were used sparingly in our series, we were unable to conclusively make this determination for accurate analysis.

## Conclusions

In conclusion, this study demonstrates that compared with a traditional approach involving separate thoracotomy and chest wall resection for P-NSCLC tumors, a "cut-in patch-out" technique may reduce outpatient narcotic requirements and appears to result in equivalent oncologic outcomes including survival rates. We therefore believe this approach warrants further investigation. Furthermore, based on this favorable experience we have more recently adapted a variation of this "cut-in patch-out" technique using non-absorbable prosthetic mesh for select patients with non P-NSCLC and chest wall invasion.

## Authors' contributions

DW, IO, and KK participated in study design. DW, IO, TB, KR, DC, and KK drafted the manuscript. All authors read and approved the final manuscript.

## Authors’ original submitted files for images

Below are the links to the authors’ original submitted files for images. Authors’ original file for figure 1 Authors’ original file for figure 2 Authors’ original file for figure 3 Authors’ original file for figure 4

## Abbreviations

NSCLC: Non small cell lung cancer
P-NSCLC: Pancoast non small cell lung cancer
