# Supplementary figures and images for: The "cut-in patch-out" technique for Pancoast tumor resections results in postoperative pain reduction: a case control study
Source: J Cardiothorac Surg. 2014 Sep 30;9:163. doi: 10.1186/s13019-014-0163-z (PMC4180969; doi:10.1186/s13019-014-0163-z)

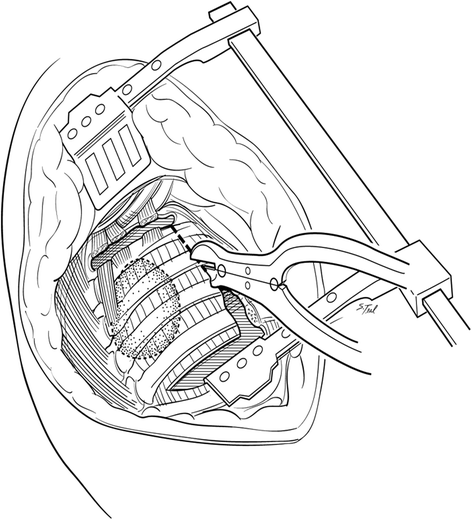

Supplement: Supplementary file 1 — Authors’ original file for figure 1 [file 13019_2014_163_MOESM1_ESM.gif]

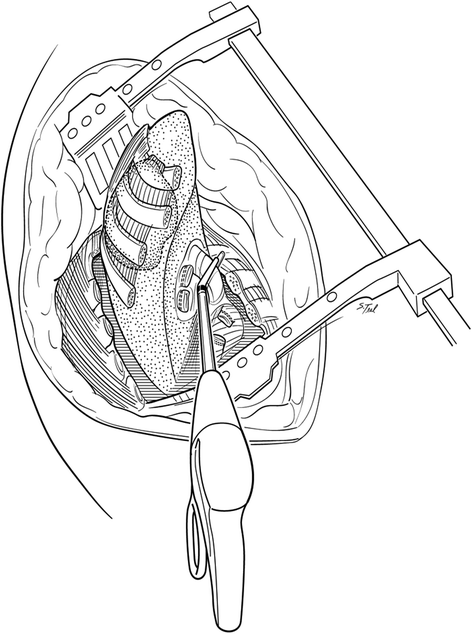

Supplement: Supplementary file 2 — Authors’ original file for figure 2 [file 13019_2014_163_MOESM2_ESM.gif]

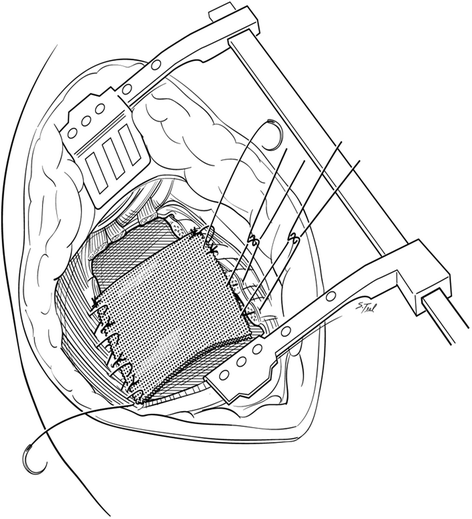

Supplement: Supplementary file 3 — Authors’ original file for figure 3 [file 13019_2014_163_MOESM3_ESM.gif]

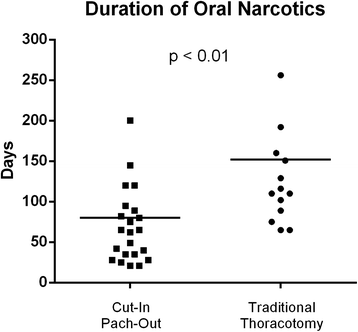

Supplement: Supplementary file 4 — Authors’ original file for figure 4 [file 13019_2014_163_MOESM4_ESM.gif]
